# Nanoparticle Formulations of siRNA: The Next Generation of Targeted Therapy for Lymphomas and Leukemias?

**DOI:** 10.1016/j.ebiom.2014.11.013

**Published:** 2014-11-20

**Authors:** Andrew Z. Wang

**Affiliations:** Laboratory of Nano- and Translational Medicine, Department of Radiation Oncology, Lineberger Comprehensive Cancer Center, Carolina Center for Cancer Nanotechnology Excellence, Carolina Institute of Nanomedicine, University of North Carolina at Chapel Hill, 101 Manning Drive, CB 7512, Chapel Hill, NC 27599-7512, USA

Many liquid tumors (lymphomas and leukemias) have characteristic genomic alterations that are critical to their disease development and progression. For example, greater than 90% of the chronic myelogenous leukemias (CML) are caused by the t(9;22)(q34;q11) or BCR/ABL translocation, whereas more than 75% of mantle cell lymphomas have the t(11,14)(q13, 32) translocation (Kurzrock et al., 2003, Li et al., 1999). Because these genomic alterations are frequently drivers of the oncogenesis process, targeted therapies that inhibit the resultant aberrant signaling pathways have achieved remarkable clinical success. Imatinib, which inhibits the ABL tyrosine kinase, is considered one of the first molecularly targeted therapies for cancer (Druker et al., 2001). A recent study suggested that patients with CML that are long-term responders to imatinib have similar survival as the general population (Gambacorti-Passerini et al., 2011). Today, many targeted therapies are in clinical use or are under clinical development for the treatment of lymphomas and leukemias (Johnston et al., 2010, Byrd et al., 2014).

Despite the success of targeted therapies, many genomic alterations are considered “undruggable”. One such example is the above-mentioned t(11,14)(q13, 32) translocation, which increases the expression of cyclin D1 (Musgrove et al., 2011). Another example of such an undruggable target is CD22ΔE12, described in a research paper by Uckun et al. in this issue of *EBioMedicine* (Uckun et al., 2014). Physiologically, CD22 negatively regulates cellular proliferation, and the CD22ΔE12 deletion results in the release of this brake. The authors demonstrated that CD22ΔE12 is associated with both pediatric and adult B-precursor acute lymphoblastic leukemias (BPL). More importantly, they showed that knockdown of CD22ΔE12 with small interfering RNA (siRNA) decreased the tumor cells' clonogenicity, suggesting that the mutation is responsible for disease progression in BPL. However, because the CD22ΔE12 mutation results in the lack of negative signaling on cellular pathways, it is an undruggable target. Despite this, the mutation is amenable to treatment by siRNA, as demonstrated by the authors.

The idea of utilizing RNA interference for the treatment of diseases is not new. As mentioned above, such a strategy can treat targets that are considered undruggable and with far-reaching potential. The key challenge in siRNA clinical translation has been drug delivery. First, siRNAs need to be preferentially delivered to diseased cells to avoid side effects to normal cells. Second, siRNA needs to enter the target cells' cytosol without degradation. Lastly, the effects of siRNA are temporary (days to weeks) and not permanent; such temporary effects may not last long enough to alter the disease course. In recent years, many siRNA delivery strategies have been developed, mostly involving the use of nanoparticles. Nanoparticles can protect the siRNA from degradation and can facilitate endosomal escape once the siRNA has been endocytosed by the target cells (Kanasty et al., 2013). However, no siRNA-based therapies have been approved for clinical use.

In the paper by Uckun et al., the investigators engineered a unique polypeptide-based nanoparticle to deliver siRNA against CD22ΔE12 (Uckun et al., 2014). The nanoparticle contains a cell-penetrating moiety that can facilitate endosomal escape of the siRNA-nanoparticle complex, solving one of the key challenges in siRNA delivery. The authors demonstrated that their CD22ΔE12 siRNA-nanoparticle can indeed knock down CD22ΔE12 and inhibit tumor cell growth. Their finding is provocative in several aspects. First, they demonstrated that siRNA can be an effective targeted therapy against genomic alterations. If there is an effective delivery vehicle, novel siRNA-based treatments can be rapidly developed to address newly discovered oncogenic mutations. Second, lymphomas and leukemias are intriguing diseases for siRNA therapy. Unlike solid tumors where nanoparticle penetration is a concern, liquid tumors are freely exposed to nanoparticle agents. Many liquid tumors also have characteristic surface biomarkers, such as CD20, that can be used for targeted drug delivery. Lastly, liquid tumor cells have rapid turnover, which makes them potentially more susceptible to the temporary effects of siRNA. Thus, this study suggests that nanoparticle siRNA formulations can be the next generation of targeted therapy in liquid tumors. However, there are several critical challenges the investigators need to overcome for the clinical translation of their technology. The authors did not evaluate the CD22ΔE12 siRNA-nanoparticle in vivo. In vivo toxicity and biodistribution are critical to a nanoparticle platform's ability as a delivery vehicle. The investigators will also need to characterize the nanoparticle's stability in vivo. Peptide-based agents are also at risk for immunogenicity, especially when the polypeptides are not native to the human body. Moreover, while the cell-penetrating moiety is beneficial for endosomal escape, it can also introduce additional systemic toxicity. The authors also did not demonstrate that these CD22ΔE12 siRNA-nanoparticles can preferentially target leukemic cells over normal cells. While normal cells may not harbor this deletion, lack of targeted delivery of the siRNA can minimize its therapeutic efficacy and increase its toxicity. Despite these limitations, this study identified CD22ΔE12 as a key genomic alteration in BPL and found that a nanoparticle formulation of siRNA against CD22ΔE12 can achieve therapeutic effects in this disease. This work highlights the potential of siRNA as targeted agents for lymphomas and leukemias.

## Disclosure

AZW is supported by R01CA178748-01 from the National Institutes of Health/National Cancer Institute and 1-U54-CA151652-01 from the National Institutes of Health Center for Nanotechnology Excellence. The author declared no conflicts of interest.
